# A113 DIAGNOSTIC ACCURACY OF COMPUTED TOMOGRAPHY ENTEROGRAPHY COMPARED TO BALLOON ASSISTED ENDOSCOPY IN CROHN’S DISEASE

**DOI:** 10.1093/jcag/gwac036.113

**Published:** 2023-03-07

**Authors:** J Cooper, T Dang, M Reeson, J Dang, L Dieleman, K Kroeker, D Baumgart, K Wong, F Peerani, S Zepeda-Gomez, S Wasilenko, F Hoentjen, E Wiebe, B Halloran

## Abstract

**Background:**

Crohn’s disease (CD) is a chronic inflammatory condition which can affect the entire gastrointestinal tract with a wide variety of potential complications which may require endoscopic or surgical interventions. Small bowel CD beyond the reach of standard endoscopy poses a diagnostic obstacle and relies on cross sectional imaging ─ such as computed tomography enterography (CTE) ─ and balloon assisted endoscopy (BAE). BAE is the current diagnostic gold standard allowing for direct mucosal visualization as well as therapeutic capabilities; however, remains limited by access, cost, and requires specialized training. Alternatively, CTE in small bowel in CD is widely available, and less invasive than BAE. The diagnostic accuracy of CTE against the gold standard of BAE remains unclear.

**Purpose:**

We aim to assess the sensitivity and specificity of CTE vs BAE in the diagnosis and evaluation of small bowel CD.

**Method:**

Patients with an established diagnosis of Crohn’s disease who underwent a CTE and a BAE within 6 months between 2011 and 2018 were reviewed. Relevant findings of active inflammation (defined by mural hyperenhancement and thickening), long-segment disease (≥ 15 cm active disease), skip-segments, number of strictures, and presence of high-grade strictures (defined on BAE as inability to traverse with scope prior to dilation and on CTE as prestenotic luminal dilation ≥3cm and fecalization) were extracted from both reports and images of CTE and BAE by two independent reviewers. Sensitivity and specificity for each finding on CTE was calculated using BAE as the gold-standard diagnostic test.

**Result(s):**

A total of 42 patients with 65 corresponding CTE and BAE were identified between 2011 and 2018. CTE was found to be most sensitive for assessing presence of active inflammation and number of strictures at 75.6% [95% CI, 60.5-87.1%] and 71.4% [95% CI, 55.4-84.3], respectively. CTE was highly specific for findings of long-segment inflammation, skip lesions, number of strictures, and high-grade stricture with a specificity of 89.3% [95% CI, 78.1-96.0], 74.1% [95%, 67.2-94.7], 100% [97.5% CI, 73.5-100], and 84.4% [95% CI, 67.2-94.7] respectively. CTE showed poor specificity for active inflammation 45.0% [23.1-68.5%], and poor sensitivity for high grade strictures 54.5% [36.4-71.9] and skip lesions 54.5% [23.4-83.3]

**Image:**

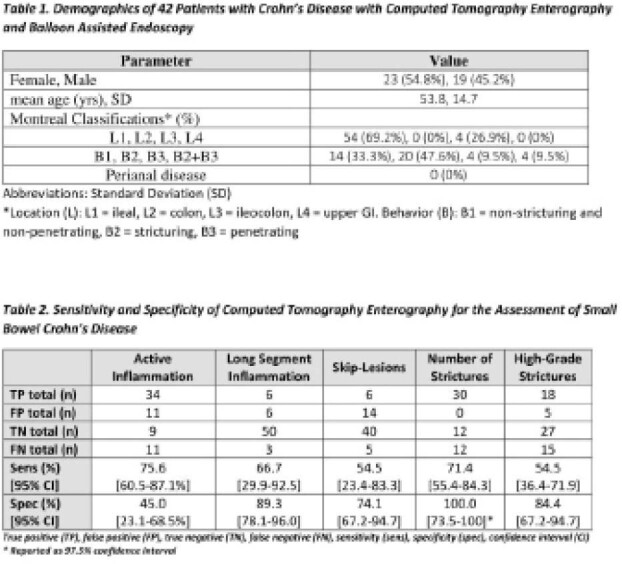

**Conclusion(s):**

CTE is relatively sensitive in detecting active inflammation and number of strictures compared to BAE, but showed suboptimal sensitivity in detecting long segment inflammation, skip lesions, and high-grade strictures. CTE showed high specificity in identification of long segment inflammation, number of strictures, and high-grade strictures, but not active inflammation overall. CTE and BAE are complementary to one another and based utilized in combination to improve diagnostic accuracy. Future directions include prospective validation prospective studies to validate the results of this study looking at a broader population of Crohn’s disease patients.

**Please acknowledge all funding agencies by checking the applicable boxes below:**

None

**Disclosure of Interest:**

None Declared

